# Prognostic significance of inferior vena cava volume defined by initial polytrauma CT-imaging: single-center experience of a level-1 trauma center

**DOI:** 10.1186/s12245-024-00752-9

**Published:** 2024-10-21

**Authors:** Hans-Jonas Meyer, Veronika Sotikova, Michael Hetz, Georg Osterhoff, Christian Kleber, Timm Denecke, Robert Werdehausen, Gunther Hempel, Manuel F. Struck

**Affiliations:** 1 Department of Diagnostic and Interventional Radiology, University Hospital Leipzig, Liebigstr.20, 04103 Leipzig, Germany; 2 Department of Orthopedics, Trauma and Plastic Surgery, University Hospital Leipzig, Liebigstr. 20, 04103 Leipzig, Germany; 3 Department of Anesthesiology and Intensive Care Medicine, University Hospital Leipzig, Liebigstr. 20, 04103 Leipzig, Germany; 4 Department of Anesthesiology and Intensive Care Medicine, Medical Faculty, Magdeburg University, Leipziger Str. 44, 39120 Magdeburg, Germany

**Keywords:** Polytrauma, Mechanical ventilation, Transfusion, Mortality, Inferior vena cava volume, Computed tomography

## Abstract

**Background:**

The significance of computed tomography (CT)-based volume measurement of the inferior vena cava (IVC) in the treatment and prognosis of trauma patients is not yet fully understood. The conflicting results that have been reported may be attributable to differences in injury severity and the use of different measurement methods, including IVC index and volumetry. The purpose of this study was to determine the relationship between IVC volume and red blood cell (RBC) transfusion and mortality in intubated trauma patients who were stable enough for initial CT imaging.

**Methods:**

A retrospective analysis was conducted on all consecutive trauma patients who underwent emergency tracheal intubation and mechanical ventilation before initial whole-body CT imaging at a level-1 trauma center over a 12-year period (2008–2019). The IVC volume was determined on initial trauma CT and included in multivariable models with demographic and diagnostic data. Associations of overall RBC transfusion, massive transfusion, 24-h mortality, and 30-day mortality were assessed using logistic regression analyses and Cox proportional hazard models.

**Results:**

A total of 438 patients (75.3% male) with a median age of 50 years, and a median injury severity score (ISS) of 26 points were included in the analysis. Most of the patients (97.5%) had suffered from blunt trauma mechanisms. Median IVC volume was 36.25 cm^3^, and RBC transfusion and massive transfusion were performed in 197 and 90 patients, respectively. The 24-h and 30-day mortality rates were 7.3% and 23.3%, respectively. VCI volume was found to be independently associated with the necessity of RBC transfusion and 24-h mortality (OR 0.98, 95% CI 0.96–0.99, *p* = 0.01 and HR 0.96, 95% CI 0.93–0.99, *p* = 0.025, respectively), while associations with massive transfusion and 30-day mortality were not statistically significant in multivariable analyses.

**Conclusion:**

Initial IVC volume may serve as a predictor of patients at risk for overall RBC transfusion requirements and 24-h mortality, suggesting the possibility of its diagnostic efficacy in short-term outcomes. Further studies are needed to confirm these findings.

## Introduction

The initial diagnostic evaluation of trauma patients includes whole-body computed tomography (CT) to detect acute life-threatening findings rapidly and reliably, which is particularly relevant in patients who have undergone emergency anesthesia, tracheal intubation and mechanical ventilation due to respiratory distress, hemodynamic parameters (shock), impaired consciousness, or severe pain conditions [1, 2].

Beyond the primary function of detecting injuries, the initial trauma CT scan allows for the assessment of other markers, including body composition parameters, bone densitometry and coronary artery calcification. This provides further valuable insights and prognostic associations [3–5].

The volume of the inferior vena cava (IVC) has been investigated as a promising imaging marker in trauma patients. In most studies examining IVC volume in trauma patients, surrogate parameters, namely the IVC index and flat IVC, were employed. These have been linked to short-term mortality [6–8], occult shock [9–11], and the need for massive transfusion [12]. However, the use of these IVC features also yielded negative results [13].

The advent of modern CT imaging analysis tools has enabled the performance of volumetry in a few seconds, thereby eliminating the need for surrogate parameters, such as the index/diameter of the IVC in a single axial slice. It seems reasonable to posit that a comprehensive IVC measurement could offer greater insights into intravascular volume status in trauma patients. This could potentially serve as a more reliable imaging marker derived from whole-body trauma CT scans.

The present study sought to identify the risk factors associated with IVC volume, as measured on initial trauma CT scans, in a cohort of severely injured trauma patients who were stable enough to undergo CT imaging. Furthermore, the relationship between IVC volume and red blood cell transfusion, massive transfusion, 24-h mortality, and 30-day mortality was investigated. We hypothesized that IVC volume is associated with these outcomes and may help identify at-risk patients early in the initial emergency CT diagnostic process.

## Materials and methods

### Patient acquisition

**Fig. 1:**
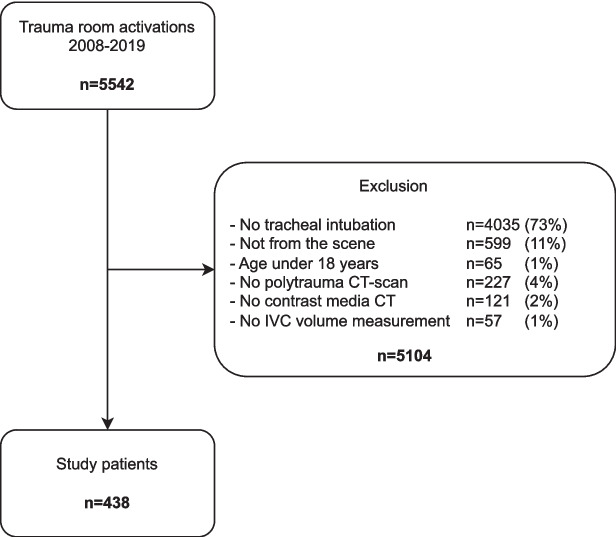
Flowchart demonstrating the recruitment of the patient cohort

Following approval by the ethics committee at the Medical Faculty of Leipzig University, Leipzig, Germany (IRB00001750, project ID 441/15ek, September 14, 2020), a retrospective analysis was conducted on consecutive trauma patients of the University Hospital Leipzig between January 2008 and December 2019. The ethics committee waived the need for informed consent, as only anonymous data were analyzed and published, and that all data were obtained in accordance with the tenets set forth in the Declaration of Helsinki. The inclusion criteria were as follows: direct admission from the scene to the emergency department (ED), emergency tracheal intubation before initial CT, performance of initial emergency CT diagnostics, and admission to the intensive care unit (ICU). Patients younger than 18 years of age, those with incomplete or missing data, and those who underwent CT imaging without contrast media were excluded from the study. The Strengthening the Reporting of Observational Studies in Epidemiology (STROBE) guidelines for cohort studies were adhered to. Figure 1 illustrates the study flowchart.

### Investigated parameters

The demographic parameters included age, sex, body mass index (BMI), and general health condition, which was defined by the American Society of Anesthesiologists classification (classes I and II were graded as healthy, and classes III or higher were graded as providing relevant pre-injury comorbidities). Injury severity was classified according to the injury severity score (ISS), impaired consciousness before tracheal intubation, as indicated by Glasgow coma scale (GCS) score of 8 points or less, and hypotension at the time of emergency department arrival, defined as systolic blood pressure (SBP) of 90 mmHg or less. The primary endpoints of the study were the transfusion requirement for red blood cells (RBCs) and the occurrence of massive transfusion (defined as the administration of ≥ 10 RBC within 24 h after admission) and all-cause mortality at 24-h and 30-days, respectively. All investigated parameters were extracted from paper-based and electronic patient charts and transformed into a tabular format for further processing, following the anonymization of personal data.

### General management

According to the standard operating procedure of the trauma center, patients with suspected severe injuries are scheduled for a whole-body trauma CT scan after evaluation by the trauma team and assessment by the trauma leader after hemodynamic stabilization. All CT scans were performed immediately after trauma team assessment before any surgical or interventional radiological procedure The distance from the Emergency Department to the CT scanners is approximately 50 m and the time for the polytrauma CT scan is 4–6 min, depending on the circulation time of the contrast medium and infrastructure management.

### Imaging technique

In this clinical setting, contrast-enhanced CT imaging was obtained using a 128-slice CT scanner (Ingenuity 128, Philips Health care, Eindhoven, The Netherlands). Iodine-based contrast medium (90 mL Imeron 400 MCT, Bracco Imaging Germany GmbH, Konstanz, Germany) was administered intravenously at a rate of 2–4.0 mL/s, in accordance with standard practice. Automatic bolus tracking was performed in the descending aorta with a trigger of 100 Hounsfield units. CT images were obtained in the late arterial phase in all cases. The typical imaging parameters were as follows: 100 kVp; 125 mAs; slice thickness, 1 mm; and pitch, 0.9. The CT scan encompassed the head to the upper thighs.

### IVC volume measurement

**Fig. 2:**
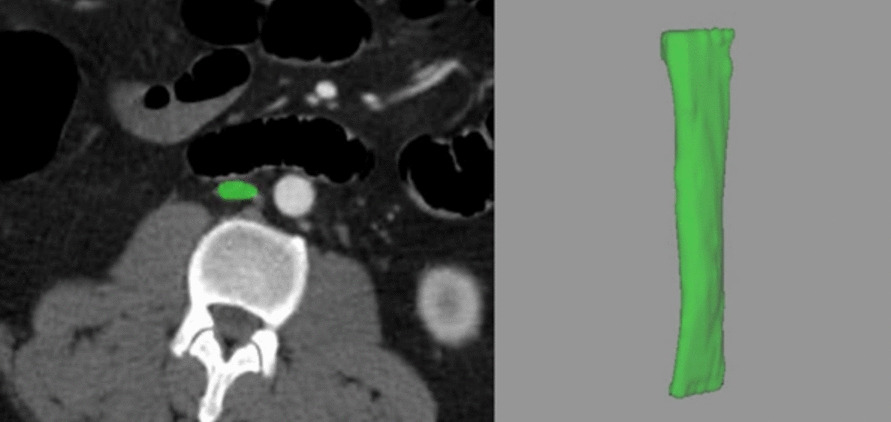
Representative 49-year-old male patient of the patient cohort. The IVC measurement is highlighted in green. The patient died after 11 days of the initial trauma. The IVC volume for this patient is 13.1 cm^3^

The IVC volume was measured semi-automatically by a trained radiologist using the picture archiving and communication system (PACS) workstation (iDS7, Sectra AB, Linköping, Sweden). The measurement was taken distally from the confluence of the iliac veins to the level of the left renal vein using the self-expanding volume tool. The volume of interest was clearly drawn inside the IVC to ensure a high reliability. The vein landmarks were previously used to provide a reliable IVC target with a better agreement between readers [14]. A comparable methodology was employed in the study by Chien et al. [14]. The reader was blinded to the patient’s clinical characteristics and outcomes. Figure 2 displays a representative measurement of one patient of the investigated patient cohort.

### Statistical analysis

The study design was primarily exploratory, and a sample size calculation was not performed due to the highly selective inclusion criteria of severely injured patients who required emergency tracheal intubation and mechanical ventilation. The categorical variables of the dataset were presented by absolute numbers and percentages, while the continuous variables were described by medians and interquartile ranges (IQR, quartile 1 and quartile 3). Sub-sequent to the testing for normal distribution via the Kolmogorov–Smirnov test, group differences were calculated using the Mann–Whitney U test, Student's t-test, and Chi-square test, when applicable. The relationship between IVC volume and the other variables was investigated using multivariable linear regression analysis. To identify independent predictors of RBC transfusion and massive transfusion, multivariable logistic regression analyses were conducted. The association between the variables and 24-h and 30-day mortality was analyzed using the Cox proportional hazard model. The results of the univariable analyses, which identified statistically significant predictors, were included in the multivariable models. The beta weights, odds ratios (OR), hazard ratios (HR), and 95% confidence intervals (CI) for IVC volume, RBC transfusion, massive transfusion, and 24-h and 30-day mortality were provided. Area under the receiver operating characteristic (AUROC) curve was provided for binary regression models. A test for multicollinearity was performed, which revealed variance inflation factor (VIF) values < 1.9 and tolerance values > 0.5, indicating that there was no relevant collinearity of the variables studied. In all instances, *p*-values < 0.05 were considered statistically significant. The statistical analysis was performed using R 4.2.2 (R Foundation for Statistical Computing, Vienna, Austria), DATAtab (DATAtab e.U., Graz, Austria) and GraphPad Prism version 10.0.2 for MacOS (GraphPad Software, Boston, Massachusetts, USA).

## Results

The study cohort consisted of 438 patients (330 males, 75.3%) with a median age of 50 years (Fig. 1, Table 1). The primary causes of injury were road traffic injuries (59%), followed by falls from height (31%), other blunt injuries (7.5%), and penetrating injuries (2.5%). The median ISS was 26 points, GCS ≤ 8 points was present in 281 patients (64.2%), and SBP ≤ 90 mmHg was present in 117 patients (26.7%). RBC transfusion within the first 24 h was required in 197 patients (45%), including 90 (20.5%) who received massive transfusion. The all-cause 24-h mortality was 7.3% (32 patients) and 30-day mortality was 22.6% (99 patients) (Table 1).

**Table 1:** Baseline characteristics of the study cohort

Parameter	All patients (*n* = 438)
Age, years; median (IQR)	50 (31 to 64)
Male sex; n (%)	330 (75.3)
BMI, kg/m^2^; median (IQR)	25 (23 to 28)
ASA ≥ III, n (%)	82 (18.7)
ISS; median (IQR)	26 (20 to 41)
GCS ≤ 8 points; n (%)	281 (64.2)
SBP ≤ 90 mmHg; n (%)	117 (26.7)
IVC volume, cm^3^; median (IQR)	36.25 (25.63 to 46.53)
RBC transfusion, n (%)	197 (45)
Massive transfusion, n (%)	90 (20.5)
24-h mortality; n (%)	32 (7.3)
30-day mortality; n (%)	99 (22.6)

*IQR* interquartile range, *BMI* body mass index, *ASA* American Society of Anesthesiologists classification, *ISS* injury severity score, *GCS* Glasgow coma scale, *SBP* systolic blood pressure, *IVC* inferior vena cava, *RBC* red blood cell unit

### Inferior vena cava volume

**Fig. 3:**
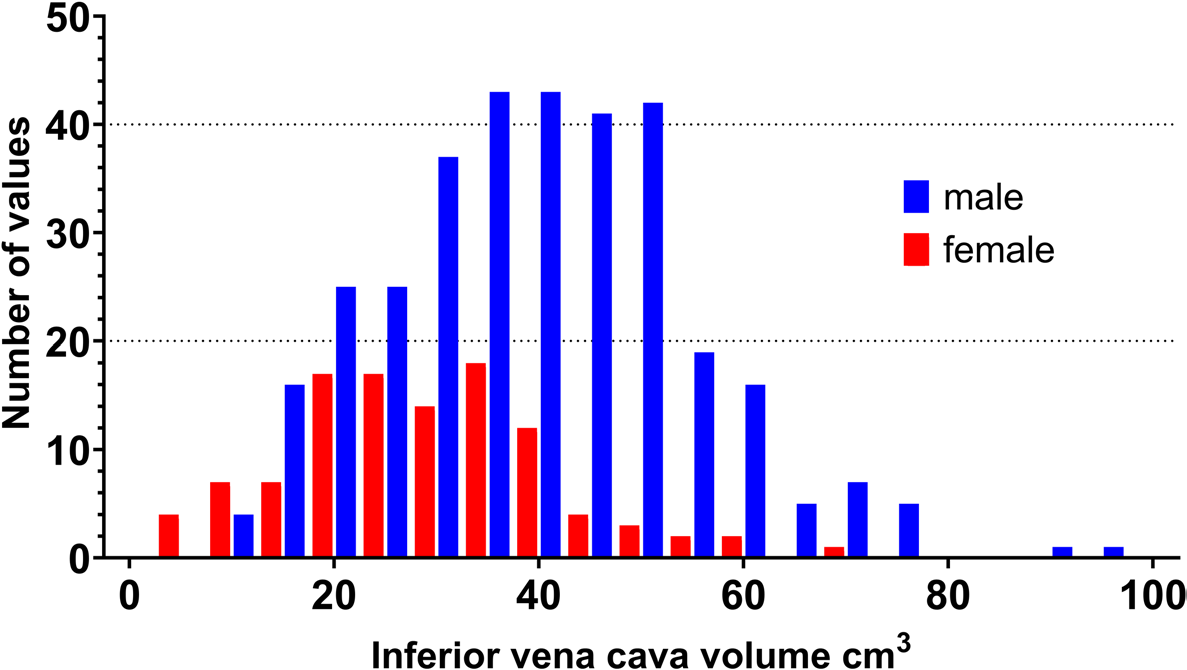
Inferior vena cava volume distribution of the study cohort

Median (IQR) IVC volume was 36.25 (25.63 to 46.53) cm^3^ (Table 1, Fig. 3). Multivariable linear regression analysis identified sex (beta 0.27, 95% CI 6.46 to 12.14, *p* < 0.001), ISS (beta -0.27, 95% CI -0.37 to -0.17, *p* < 0.001), and SBP ≤ 90 mmHg (beta -0.19, 95% CI -9.72 to -3.19, *p* < 0.001) as statistically significant associations with IVC volume (Table 2).

**Table 2:** Linear regression analyses of associations with inferior vena cava volume

Parameter	Univariable beta	95% CI	p value	Multivariable beta	95% CI	*p*-value
Age	-0.06	-0.11 to 0.03	0.226			
Sex (1-male)	-0.31	-13.73 to -7.48	** < 0.001**	0.27	6.46 to 12.14	** < 0.001**
BMI	0.01	-0.32 to 0.41	0.793			
ASA ≥ III	-0.04	-4.99 to 2.23	0.45			
ISS	-0.39	-0.48 to -0.3	** < 0.001**	-0.27	-0.37 to -0.17	** < 0.001**
GCS ≤ 8 points	-0.08	-5.31 to 0.57	0.112			
SBP ≤ 90 mmHg	-0.36	-15.09 to -9.13	** < 0.001**	-0.19	-9.72 to -3.19	** < 0.001**

*Beta* standardized coefficient, *CI* confidence interval, *BMI* body mass index, *ASA* American Society of Anesthesiologists classification, *ISS* injury severity score, *GCS* Glasgow coma scale, *SBP* systolic blood pressure; bold numbers indicate statistical significance (*p* < 0.05)

### Red blood cell transfusion

Patients requiring red blood cell transfusion had significantly lower IVC volumes than patients without transfusion (Table 3). Univariable logistic regression analysis identified statistically significant associations ISS, GCS ≤ 8 points, SBP ≤ 90 mmHg, and IVC volume (Table 4a). Multivariable analysis confirmed ISS (OR 1.09, 95% CI 1.06 to 1.11, *p* < 0.001), IVC volume (OR 0.98, 95% CI 0.96 to 0.99, *p* = 0.01), and SBP ≤ 90 mmHg (OR 1.83, 95% CI 1.01 to 3.31, *p* < 0.001), as statistically significant associations, while GCS ≤ 8 points was not significantly associated.

**Table 3:** Inferior vena cava volumes in investigated outcome categories

Outcome	IVC volume, cm^3^ Yes	IVC volume, cm^3^ No	*p*-value
Red blood cell transfusion	30.6 (21.1 to 41.5)	40.1 (32.2 to 49.9)	** < 0.001**
Massive transfusion	24.9 (17.3 to 37.67)	38.8 (29.7 to 47.78)	** < 0.001**
24-h mortality	21.95 (13.55 to 29.88)	37.3 (27.2 to 47.13)	** < 0.001**
30-day mortality	29.6 (20.8 to 40.05)	38.1 (28.8 to 47.85)	** < 0.001**

*IVC* inferior vena cava volume (median (IQR); bold numbers indicate statistical significance (*p* < 0.05)

**Table 4:** Logistic regression analyses of associations with red blood cell transfusion and massive transfusion within the first 24 h after admission

Parameter	Univariable OR	95% CI	*p* value		Multivariable OR	95% CI		*p*-value
**a). Red blood cell transfusion (*n* = 197 patients)**								
Age	1	0.99 to 1.01	0.799					
Sex (1 = male)	1.37	0.89 to 2.11	0.153					
BMI	1.04	0.99 to 1.1	0.082					
ASA ≥ III	1.07	0.67 to 1.73	0.772					
ISS	1.11	1.08 to 1.13	** < 0.001**		1.09	1.06 to 1.11		** < 0.001**
GCS ≤ 8 points	2.28	1.52 to 3.41	** < 0.001**		1.02	0.63 to 1.67		0.929
SBP ≤ 90 mmHg	6.26	3.87 to 10.12	** < 0.001**		1.83	1.01 to 3.31		** < 0.001**
IVC volume, cm^3^	0.95	0.94 to 0.97	** < 0.001**		0.98	0.96 to 0.99		**0.01**
**b). Massive transfusion (*n* = 90 patients)**								
Age	0.99	0.98 to 1	0.125					
Sex (1 = male)	1.29	0.77 to 2.16	0.333					
BMI	1.02	0.96 to 1.08	0.464					
ASA ≥ III	0.9	0.49 to 1.64	0.733					
ISS	1.11	1.08 to 1.13	** < 0.001**	1.07		1.05 to 1.1	** < 0.001**	
GCS ≤ 8 points	2.72	1.56 to 4.75	** < 0.001**	0.99		0.48 to 2.03	0.976	
SBP ≤ 90 mmHg	14.99	8.73 to 25.74	** < 0.001**	4.94		2.6 to 9.39	** < 0.001**	
IVC volume, cm^3^	0.94	0.92 to 0.96	** < 0.001**	0.99		0.96 to 1.01	0.19	

*OR* odds ratio, *CI* confidence interval, *BMI* body mass index, *ASA* American Society of Anesthesiologists classification, *ISS* injury severity score, *GCS* Glasgow coma scale, *SBP* systolic blood pressure, *IVC* inferior vena cava; bold numbers indicate statistical significance (*p* < 0.05). Area under the receiver operating characteristic (AUROC) curve for red blood cell transfusion a) 0.833, and for massive transfusion b) 0.878, respectively

### Massive transfusion

Patients who underwent massive transfusion had significantly lower IVC volumes than those who did not (Table 3). Univariable logistic regression analysis revealed statistically significant associations of massive transfusion with ISS, GCS ≤ 8 points, SBP ≤ 90 mmHg, and IVC volume (Table 4b). Multivariable analysis confirmed ISS (OR 1.07, 95% CI 1.05 to 1.1, *p* < 0.001) and SBP ≤ 90 mmHg (OR 4.94, 95% CI 2.6 to 9.39, *p* < 0.001) as statistically significant associations, while GCS ≤ 8 points and IVC volume were not significantly associated.

### 24-h mortality

Inferior vena cava volumes were significantly lower in non-survivors compared with survivors at 24 h (Table 3). In the Cox proportional hazards model, statistically significant univariable associations with 24-h mortality were age, ASA classification ≥ III, ISS, GCS ≤ 8 points, SBP ≤ 90 mmHg (Table 5a). Adjusted for multiple predictors, 24-h mortality was significantly associated with ISS (HR 1.05, 95% CI 1.02 to 1.07, *p* < 0.001), ASA classification ≥ III (HR 4.31, 95% CI 1.76 to 10.55, *p* = 0.001), IVC volume (HR 0.96, 95% CI 0.93 to 1, *p* = 0.025), and SBP ≤ 90 mmHg (HR 2.59, 95% CI 1 to 6.7, *p* = 0.049), while GCS ≤ 8 points revealed no significant association.

**Table 5:** Cox proportional hazard models of associations with 24-hour and 30-day mortality

Parameter	Univariable HR	95% CI	*p* value	Multivariable HR	95% CI	*p*-value
**a). 24-hour mortality (*n* =32 patients)**						
Age	1.03	1.01 to 1.05	**0.001**	1	0.97 to 1.03	0.985
Sex (1=male)	0.82	0.37 to 1.84	0.637			
BMI	0.99	0.9 to 1.09	0.845			
ASA ≥III	5.95	2.83 to 12.05	**<0.001**	4.31	1.76 to 10.55	**0.001**
ISS	1.08	1.05 to 1.1	**<0.001**	1.05	1.02 to 1.07	**<0.001**
GCS ≤8 points	19.34	2.61 to 143.13	**0.004**	4.87	0.63 to 37.59	0.129
SBP ≤90 mmHg	12.19	5.11 to 29.09	**<0.001**	2.59	1 to 6.7	**0.049**
IVC volume, cm^3^	0.92	0.89 to 0.95	**<0.001**	0.96	0.93 to 0.99	**0.025**
**b). 30-day mortality (*n* = 99 patients)**						
Sex (1=male)	1.01	0.64 to 1.58	0.973			
BMI	1.05	1 to 1.1	**0.035**	1.04	0.99 to 1.1	0.12
ASA ≥III	3.01	2.01 to 4.51	**<0.001**	3.42	1.95 to 6.02	**<0.001**
ISS	1.07	1.05 to 1.08	**<0.001**	1.05	1.04 to 1.07	**<0.001**
GCS ≤8 points	8.23	3.82 to 17.73	**<0.001**	3.37	1.52 to 7.49	**0.003**
SBP ≤90 mmHg	5.35	3.6 to 7.96	**<0.001**	2	1.25 to 3.22	**0.004**
IVC volume, cm^3^	0.96	0.95 to 0.98	**<0.001**	0.99	0.98 to 1.01	0.219

*HR* hazard ratio, *CI* confidence interval, *BMI* body mass index, *ASA* American Society of Anesthesiologists classification, *ISS* injury severity score, *GCS* Glasgow coma scale, *SBP* systolic blood pressure, *IVC* inferior vena cava; bold numbers indicate statistical significance (*p* <0.05). Area under the receiver operating characteristic (AUROC) curve for 24-hour mortality a) 0.922 and for 30-day mortality b) 0.884.

Including only ISS, SBP ≤ 90 mmHg, and IVC volume into the analysis to provide at least 10 events per variable, all statistically significant associations remained significant (ISS, HR, 1.04, 95**%** CI 1.02 to 1.07, *p* < 0.001; SBP ≤ 90 mmHg, HR 3.3, 95% CI 1.23 to 8.84, *p* = 0.017; and IVC volume, HR, 0.97, 95% CI 0.94 to 0.99, *p* = 0.027, respectively).

### 30-day mortality

Patients who died within 30 days of admission had significantly lower IVC volumes than survivors (Table 3). Univariable analysis of 30-day mortality revealed statistically significant associations with age, BMI, ASA classification ≥ III, ISS, GCS ≤ 8 points, SBP ≤ 90 mmHg, and IVC volume (Table 5.b). Multivariable analysis confirmed ASA classification ≥ III (HR 3.42, 95% CI 1.95 to 6.02, *p* < 0.001), ISS (HR 1.05, 95% CI 1.04 to 1.07, *p* < 0.001), GCS ≤ 8 points (HR 3.37, 95% CI 1.52 to 7.49, *p* = 0.003), and SBP ≤ 90 mmHg (HR 2, 95% CI 3.22 to 1.1, *p* = 0.004) as statistically significant associations, while BMI and IVC volume were not significantly associated.

## Discussion

The present analysis suggest that IVC volume was independently associated with RBC transfusion and 24-h mortality. Although univariable analyses revealed associations of IVC volume with massive transfusion and 30-day mortality, they both were statistically not significant after adjustment for multiple predictors.

Previous studies have tried to elucidate the prognostic relevance of IVC volume in trauma patients [6–14]. Among them, Johnson et al. identified 30 patients with flat IVC in their patient cohort of 161 trauma patients [8]. They reported an inverse correlation of IVC ratio with initial bicarbonate, hemoglobin, and base excess, and a direct correlation with ISS. In the multivariable analysis adjusted for age, ISS, and presence of severe head injury, patients with flat IVC were 8.1 times (95% CI 1.5–42.9) more likely to die, compared with the nonexposed cohort [8].

As initially mentioned, not all studies could show such clear results with IVC assessment. Radomski et al. could not find an association between the IVC index and clinical outcomes in a patient sample of 272 trauma patients [14]. There was no association between IVC size and the need for urgent operation, angiography, emergent transfusion, hospital length of stay, or mortality [14].

These inconsistent findings comprising 12 studies with a total of 1706 trauma patients were pooled and interpreted in a meta-analysis by Kim et al., published in 2022 [15]. Flat IVC provided an acceptable diagnostic accuracy to predict the development of shock (AUC 0.78), whereas only poor accuracy for mortality prediction was found (AUC 0.60) [15]. It was concluded that the included studies provided substantial heterogeneity regarding different levels of the measurement for the IVC diameter. Moreover, these studies used only a ratio of the IVC to determine the flat IVC and no volumetry [15].

One study by Chien et al. including 236 trauma patients was conducted using IVC volumetry for prediction of the need for massive transfusion and short-term mortality [14]. The authors reported an excellent inter-reader agreement (intra-class correlation 0.96) and divided the patient cohort into four groups representing the four quartiles of IVC volume measurement. The key finding of this study was an association of the IVC volume with massive transfusion, which was performed in 18 patients (7.6%) in contrast to the three-fold rate of the present study of 90 patients (20.5%). The reported sensitivity to correctly predict a patient with a need for transfusion was 66.7% and a specificity of 77.9% using a IVC volume threshold < 15.08 cm^3^ (first quartile)[14]. Of note, the proportion of female patients was highest in this quartile. In the present study, the corresponding volume of quartile one was considerably higher (< 25.63 cm^3^) which was consequently also the case in quartiles two (25.63 to 36.25 cm^3^ vs. 15.08 to 20.6 cm^3^ [14]), three (36.25 to 46.53 cm^3^ vs. 20.6 to 28.29 cm^3^ [14]), and four (> 46.53 cm^3^ vs. > 28.29 cm^3^ [14]), respectively. One possible reason for these different quartile ranges might be the ethnicity of the included patients and race-dependent differences, but data regarding body height and weight of the study cohort of Chien et al. have not been reported [16, 17]. We found significantly lower IVC volumes in female patients, which should be considered when interpreting the data.

Contrary to the present study, the study of Chien et al. could not demonstrate an association between IVC volume and 24-h or 30-day mortality (OR 0.92, 95% CI 0.84–1.0, and OR 0.98, 95% CI 0.94–1.02, respectively). The most plausible reasons for the different results might be the lower injury severity and mortality rates of the two cohorts (Chien et al., median ISS 13 points, 24-h mortality 4.7%, and 30-day mortality 11.4%) [14].

Besides CT images for measurement of IVC, there are promising results of the ability to assess the IVC with sonography [18]. The study group around Kim et al. could show in another large meta-analysis that the ultrasonographic measurement of the variation in diameter of the IVC provides a favourable diagnostic accuracy for predicting fluid responsiveness in critically ill patients [18]. However, sonography may be prone to increased inter-reader variability and other inherent limitations [19]. The potential benefits of CT in measuring the entire volume of the IVC, as opposed to only a small area of the vessel, as would be the case with sonography should be acknowledged.

A crucial element of CT is the instruction to patients to pause their breathing after a full inspiration, as this technique enhances the image quality. The act of inhaling reduces thoracic pressure and increases venous return [18]. This results in a reduction in the diameter of the IVC, which may render the CT measurement representative of the minimum diameter or volume of the IVC in comparison to sonography [17]. It is reasonable to hypothesize that this effect may be less pronounced in the present cohort of mechanically ventilated patients, and this should be considered in any subsequent analysis. Nevertheless, there is a paucity of studies that have been conducted to compare the IVC volume of mechanically ventilated trauma patients to that of less injured trauma patients.

The present study may be another contribution to the usefulness of prognostic imaging markers derived from the initial trauma CT. The potential implications of IVC volume as a CT-derived imaging marker of intravascular volume status may prove valuable in indicating shock independently of the availability of hemodynamic data, which may be biased from long-term medication (e.g., beta-blockers), anesthetic agents, vasopressors, and fluid resuscitation. These associations need to be investigated in further prospective studies.

The present study has limitations. First, single-center retrospective studies may be susceptible to potential inherent biases, although the imaging analysis was conducted in a blinded manner with respect to the clinical data. Secondly, the study is subject to a selection bias, as it only includes patients who underwent emergency tracheal intubation and mechanical ventilation, and who underwent whole-body CT. The necessity for advanced airway management and mechanical ventilation during trauma resuscitation serves as a straightforward and pragmatic indicator of significant injury severity. As a result of this limitation, the cohort was homogeneous with high overall injury severity and mortality rates. Furthermore, it can be postulated that varying levels of positive end-expiratory pressure and different fluid resuscitation volumes (including transfusion units) may have resulted in erroneous IVC measurements, which must be taken into consideration when interpreting the present findings. Thirdly, although the IVC volume measurement is a semiquantitative imaging analysis, it is not possible to rule out the possibility of investigator-related bias entirely. Fourth, traumatic injuries have the potential to result in an erroneous measurement of the IVC. For example, the presence of large retroperitoneal hemorrhage, abdominal compartment syndrome, or injuries to the IVC itself could result in a false negative small volume. However, in the present cohort, no relevant findings were identified by the reader, and therefore, the influence of this potential bias seems minimal. Additionally, the dataset revealed notable sex-specific discrepancies in IVC volumes, with a disproportionate distribution of male and female patients. This aspect must be considered when designing future studies.

## Conclusion

In conclusion, initial polytrauma CT imaging-defined IVC volume may serve as a predictor to identify patients at risk for blood transfusion requirements and 24-h mortality. Further validation studies, including adjustment of fluid resuscitation volumes and vasopressor dosing, are needed to confirm these findings.

## Data Availability

The datasets used and/or analyzed during the current study are available from the corresponding author on reasonable request.
